# Supplementary figures and images for: Failure of colonization following gut microbiota transfer exacerbates DSS-induced colitis
Source: Gut Microbes. 2025 Jan 15;17(1):2447815. doi: 10.1080/19490976.2024.2447815 (PMC11740679; doi:10.1080/19490976.2024.2447815)

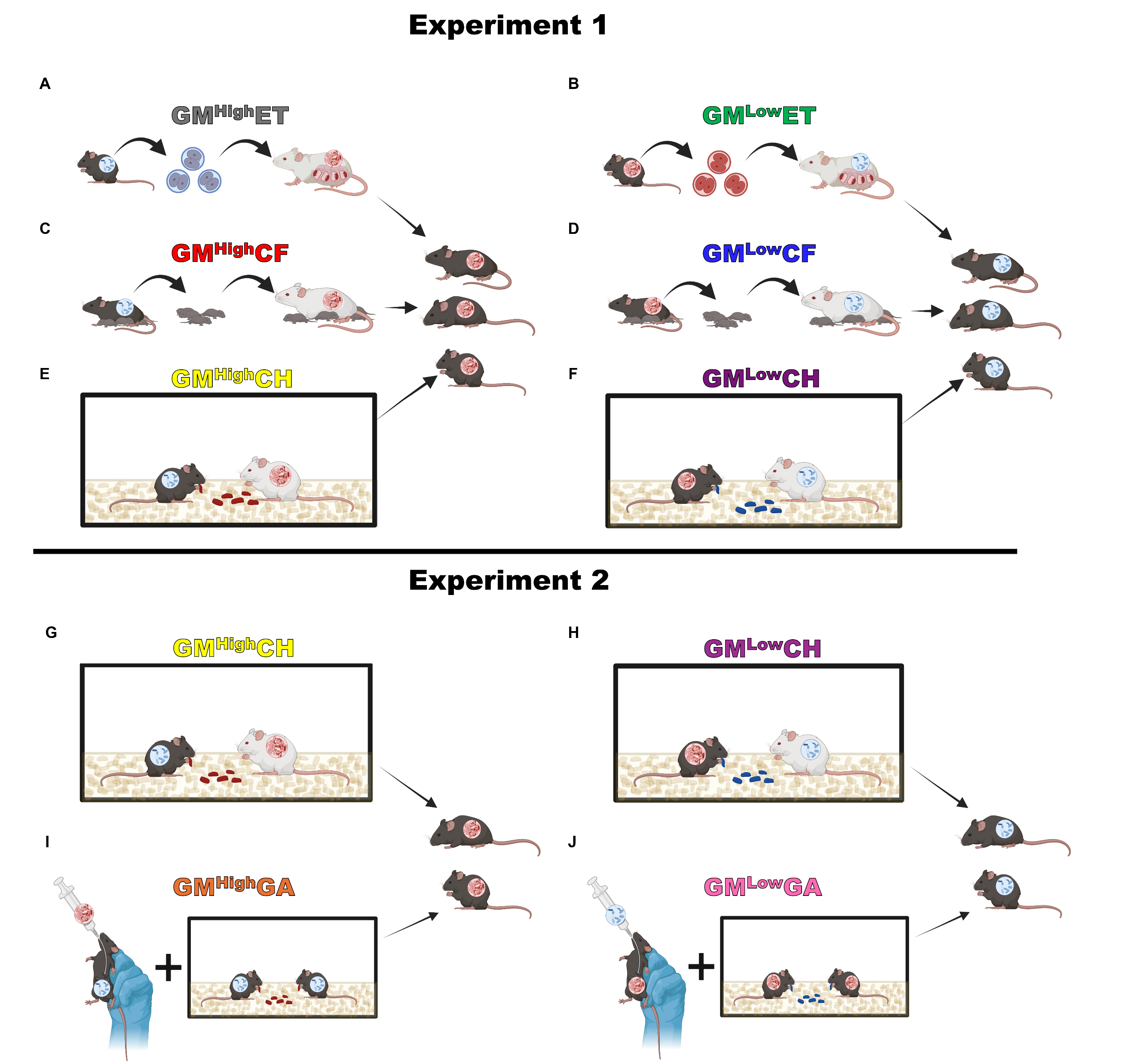

Supplement: Supplemental Material [file KGMI_A_2447815_SM5313.zip › KGMI_A_2447815/kgmi-s-2024-1834-20241224163914/Sup Fig 1.tif]

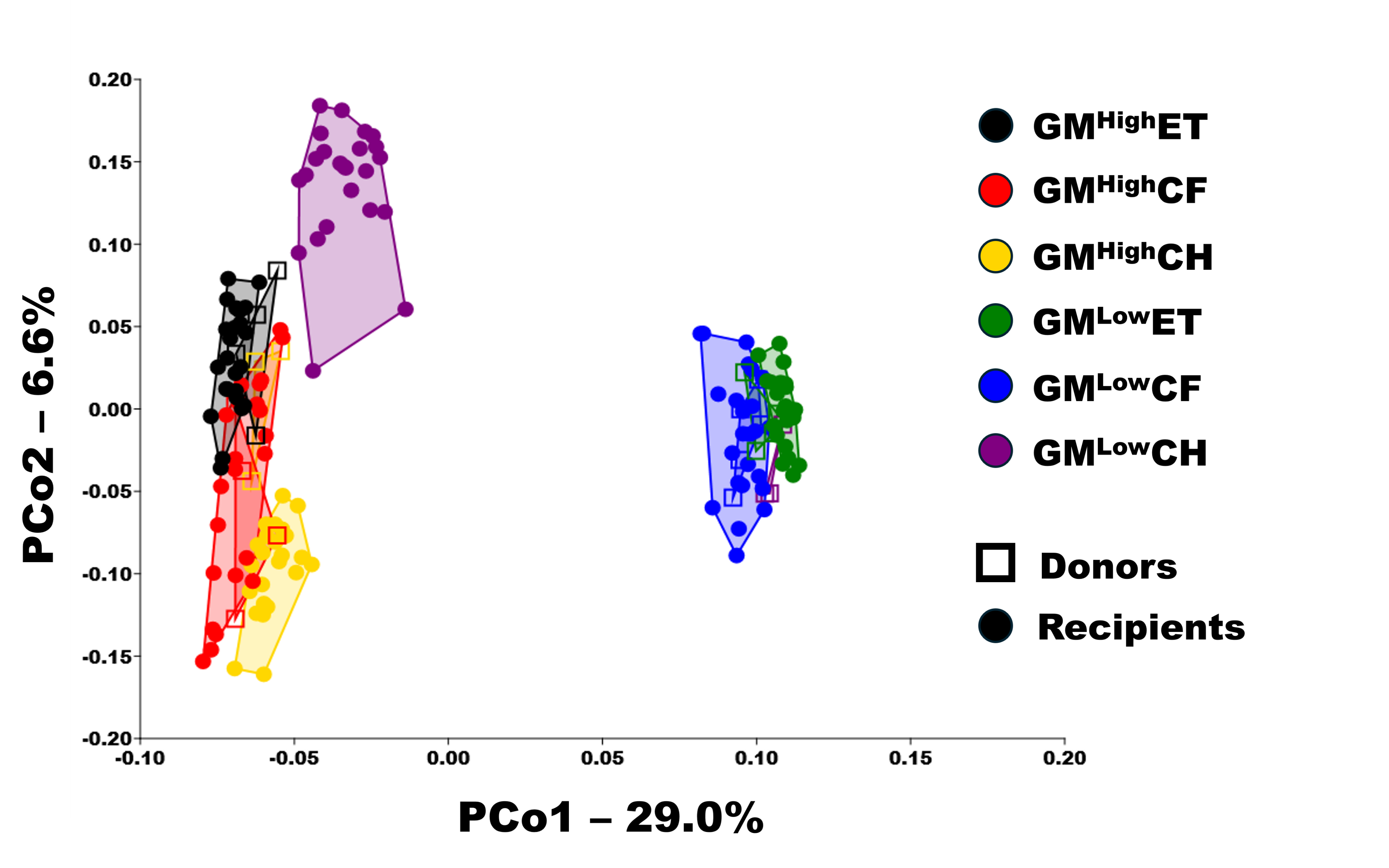

Supplement: Supplemental Material [file KGMI_A_2447815_SM5313.zip › KGMI_A_2447815/kgmi-s-2024-1834-20241224163914/Sup Fig 2.tif]

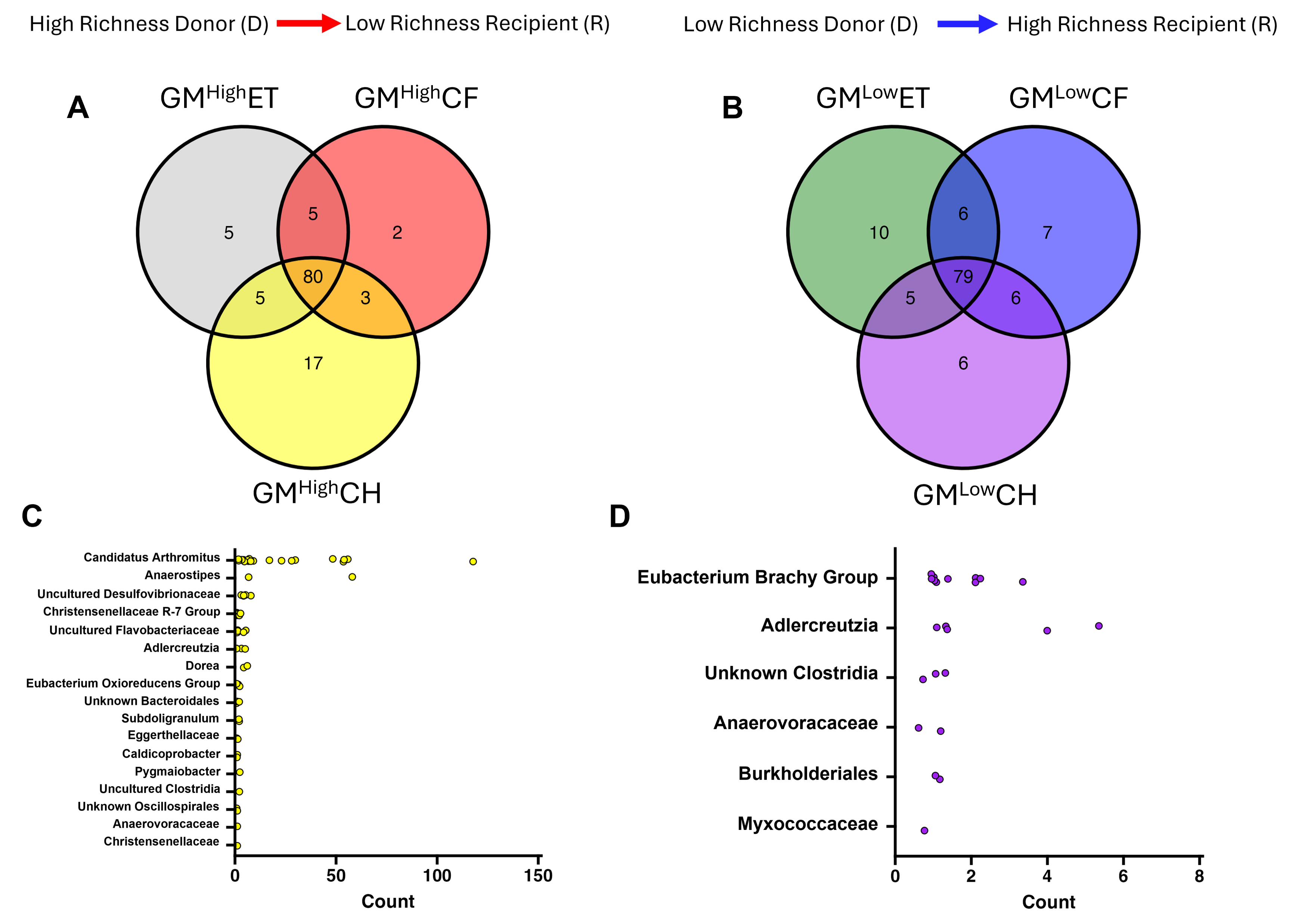

Supplement: Supplemental Material [file KGMI_A_2447815_SM5313.zip › KGMI_A_2447815/kgmi-s-2024-1834-20241224163914/Sup Fig 3.tif]

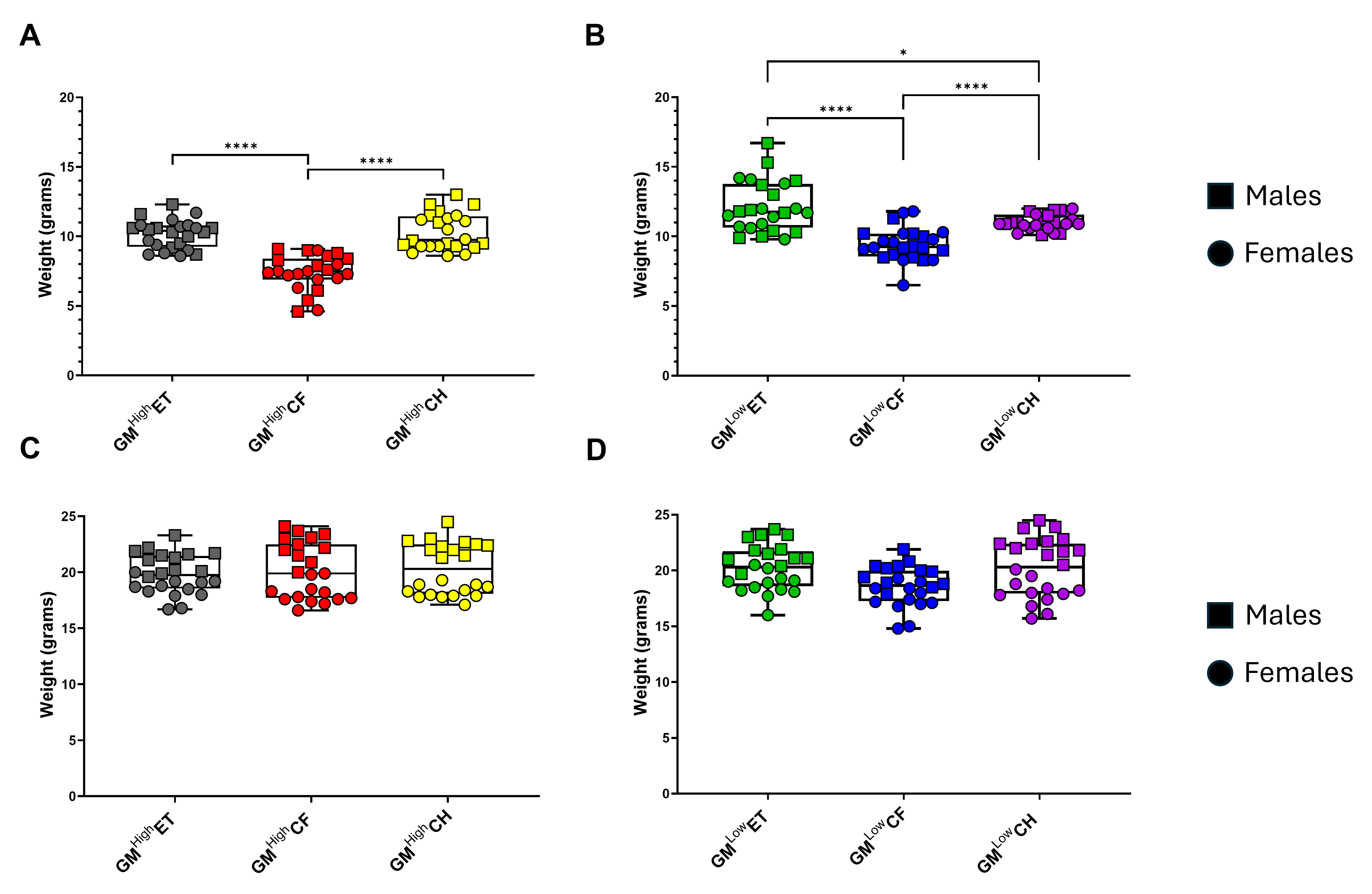

Supplement: Supplemental Material [file KGMI_A_2447815_SM5313.zip › KGMI_A_2447815/kgmi-s-2024-1834-20241224163914/Sup Fig 4.tif]

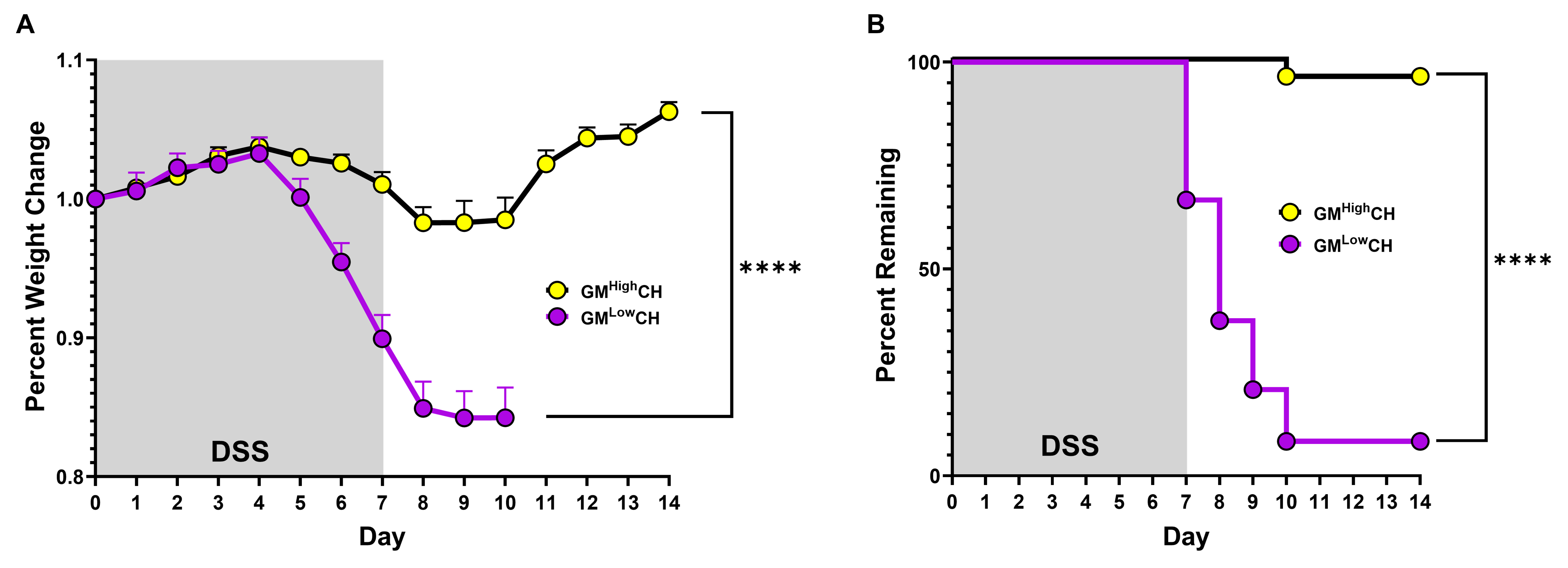

Supplement: Supplemental Material [file KGMI_A_2447815_SM5313.zip › KGMI_A_2447815/kgmi-s-2024-1834-20241224163914/Sup Fig 5.tif]

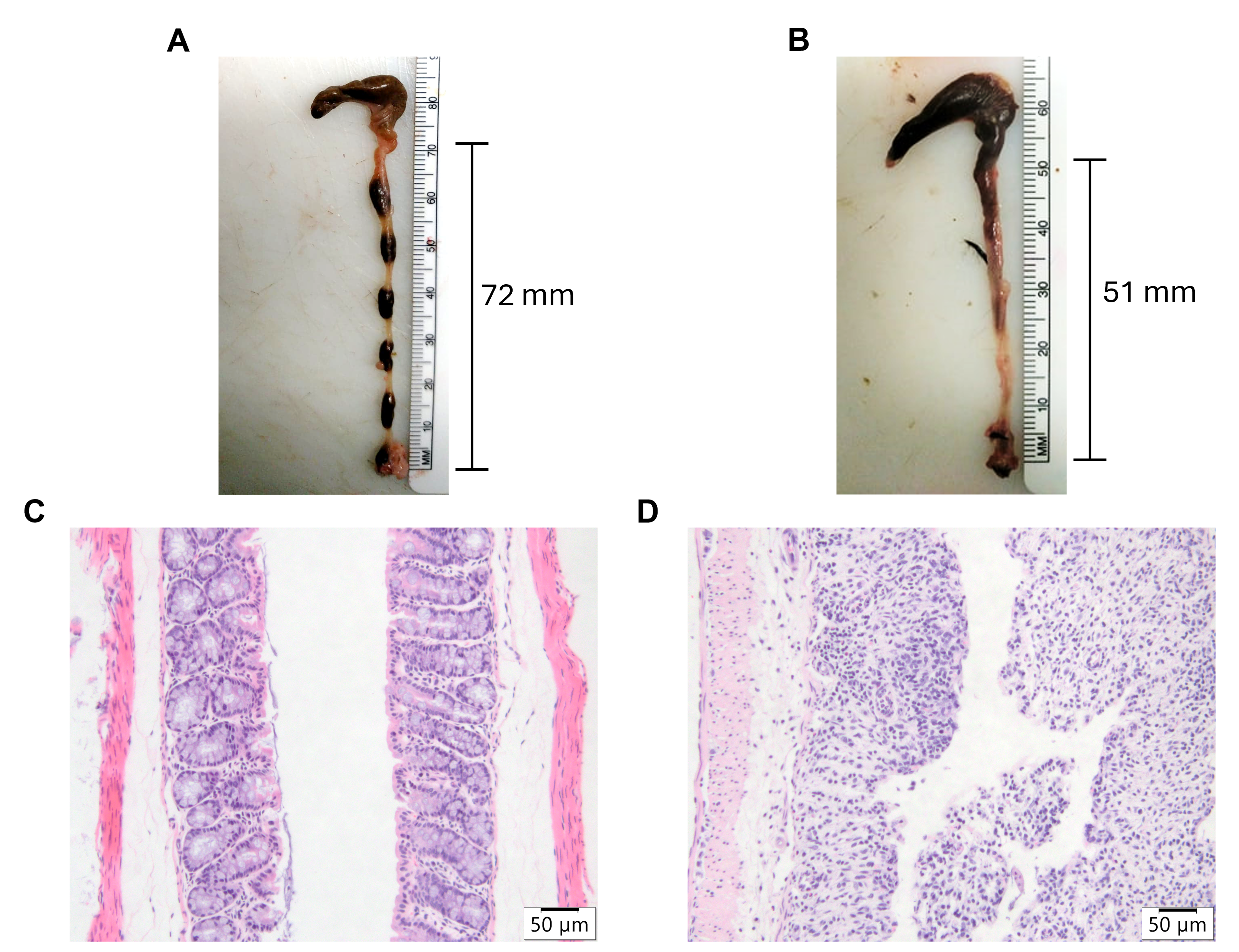

Supplement: Supplemental Material [file KGMI_A_2447815_SM5313.zip › KGMI_A_2447815/kgmi-s-2024-1834-20241224163914/Sup Fig 6.tif]

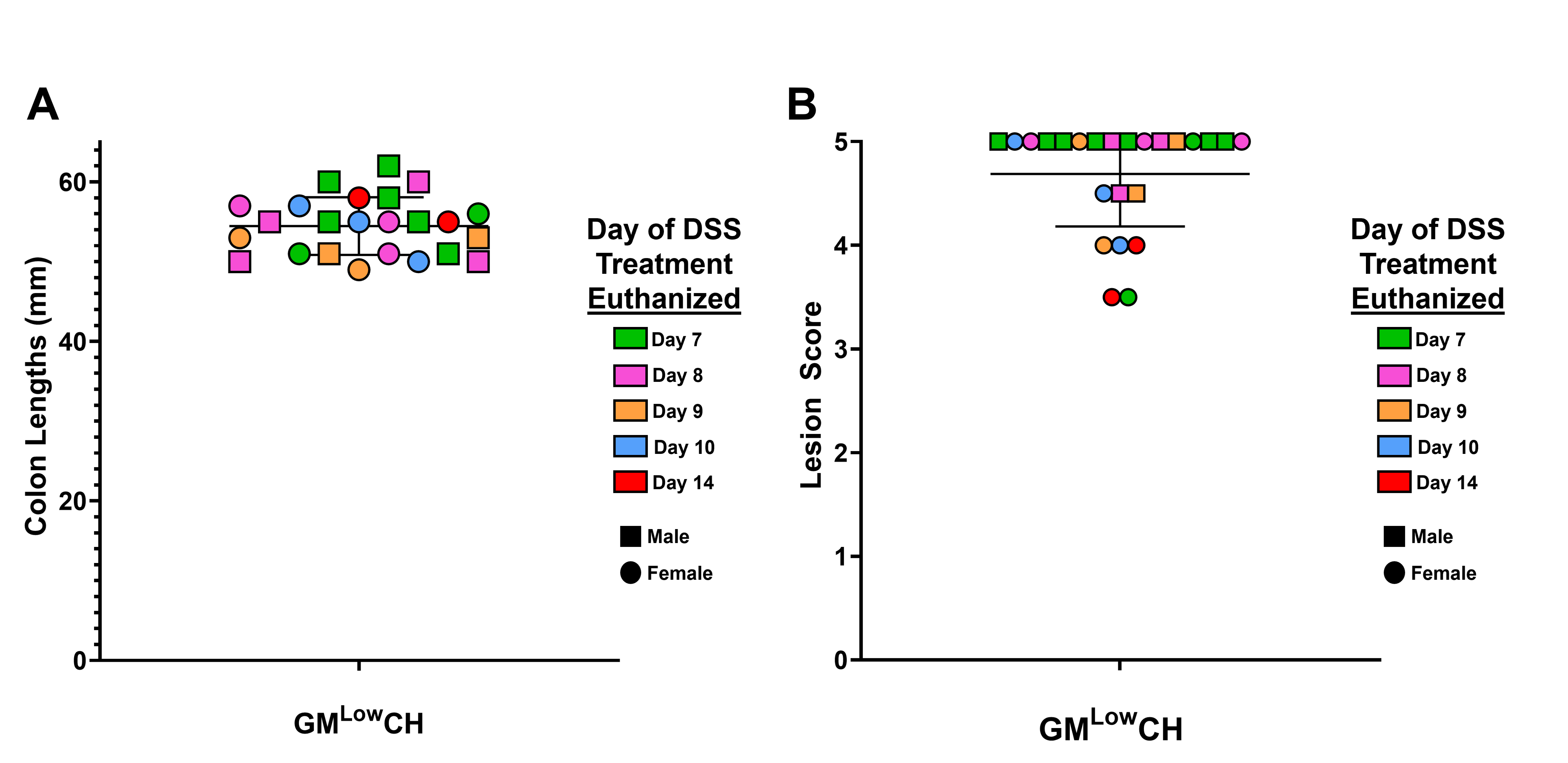

Supplement: Supplemental Material [file KGMI_A_2447815_SM5313.zip › KGMI_A_2447815/kgmi-s-2024-1834-20241224163914/Sup Fig 7.tif]

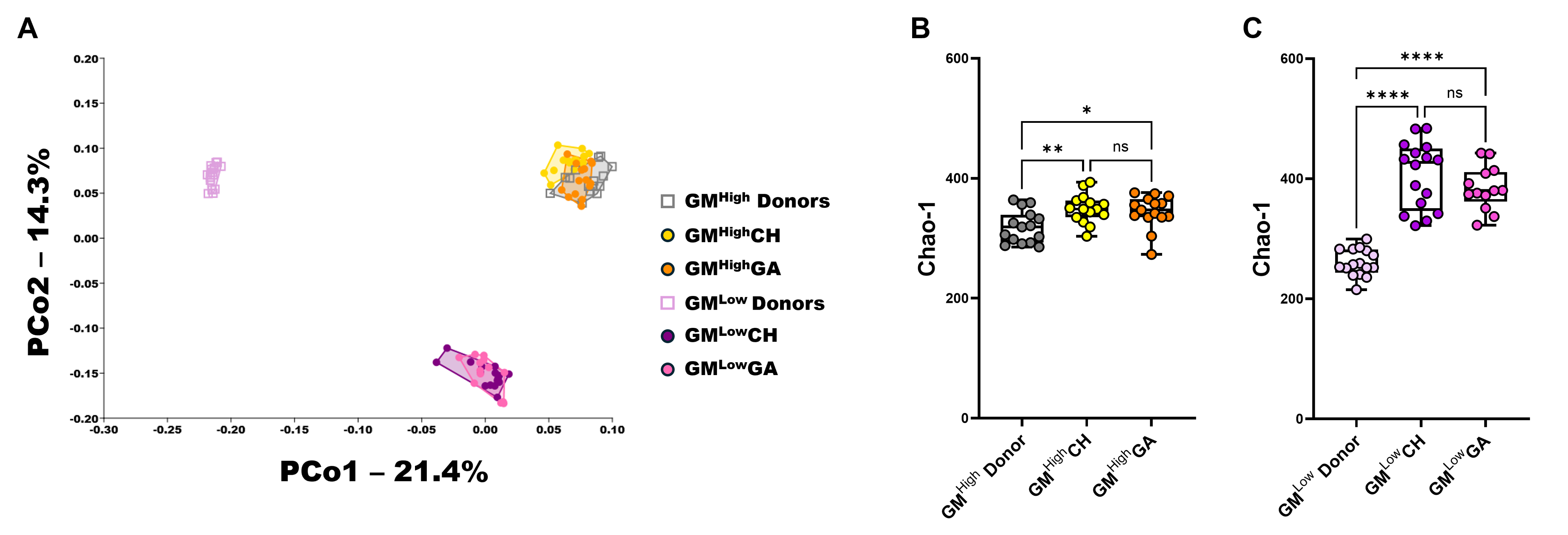

Supplement: Supplemental Material [file KGMI_A_2447815_SM5313.zip › KGMI_A_2447815/kgmi-s-2024-1834-20241224163914/Sup Fig 8.tif]
